# Shear Wave Dispersion in Chronic Liver Disease: From Physical Principles to Clinical Usefulness

**DOI:** 10.3390/jpm13060945

**Published:** 2023-06-02

**Authors:** Matteo Garcovich, Mattia Paratore, Maria Elena Ainora, Laura Riccardi, Maurizio Pompili, Antonio Gasbarrini, Maria Assunta Zocco

**Affiliations:** 1 Medicina Interna e Gastroenterologia, CEMAD Digestive Disease Center, Fondazione Policlinico Universitario A. Gemelli IRCCS, Largo A. Gemelli 8, 00168 Rome, Italy; matteo.garcovich@policlinicogemelli.it (M.G.);; 2 Università Cattolica del Sacro Cuore, Largo A. Gemelli 8, 00168 Rome, Italy; 3 Medicina Interna e del Trapianto di Fegato, Fondazione Policlinico Universitario A. Gemelli IRCCS, Largo A. Gemelli 8, 00168 Rome, Italy

**Keywords:** multiparametric ultrasound, viscosity, share wave dispersion, inflammation, steatosis, fibrosis, chronic liver disease, elastography

## Abstract

The development of new applications in ultrasound (US) imaging in recent years has strengthened the role of this imaging technique in the management of different pathologies, particularly in the setting of liver disease. Improved B-mode imaging (3D and 4D), contrast-enhanced US (CEUS) and especially US-based elastography techniques have created the concept of multiparametric ultrasound (MP-US), a term borrowed from radiological sectional imaging. Among the new elastography techniques, shear wave dispersion is a newly developed imaging technology which enables the assessment of the shear waves’ dispersion slope. The analysis of the dispersion qualities of shear waves might be indirectly related to the tissue viscosity, thus providing biomechanical information concerning the pathologic state of the liver such as necroinflammation. Some of the most recent US devices have been embedded with software that evaluate the dispersion of shear waves/liver viscosity. In this review, the feasibility and the clinical applications of liver viscosity are reviewed based on the preliminary findings of both animal and human studies.

## 1. Introduction

Chronic liver disease (CLD) comprises different pathologic conditions that impair the liver parenchyma over time and can lead to compensated advanced chronic liver disease, cirrhosis, and hepatocellular carcinoma [1]. With 844 million people affected, 2 million deaths annually, and a high morbidity rate that has a significant impact on the quality of life and the economic system, CLD is an extremely critical problem for public health [2]. The aetiologies of CLD vary according to geographical areas. With a global prevalence of 25%, non-alcoholic fatty liver disease (NAFLD) is the primary cause of CLD in Europe and the United States, followed by alcoholic liver disease (ALD), whereas in Asia and sub-Saharan Africa, chronic hepatitis B and C infections continue to be major aetiological factors [3,4,5]. Given the changes in lifestyle, particularly in China over the past two decades, it is likely that a nonviral disease such as NAFLD will become the predominant cause of CLD worldwide in the future [6,7,8].

Steatosis, necroinflammation, and consequent fibrosis are the molecular mechanisms which sustain the progression from reversible stages of disease to irreversible advanced fibrosis, cirrhosis, and its complications [9,10,11]. Consequently, it is essential to individuate and quantify these pathological changes when diagnosing and assessing CLD, particularly in reversible phases [12]. A liver biopsy is considered the gold standard for fibrosis evaluation and staging, as well as for the categorization of steatosis, necrosis, and inflammatory activity [13,14,15,16]. However, the invasiveness, costs, risk of life-threatening complications, sampling bias, variability among pathology reports, and the limited applicability for longitudinal follow up prompted the development of non-invasive approaches [17]. Recently, magnetic resonance elastography (MRE) for fibrosis assessment and magnetic resonance imaging with proton density fat fraction (MRI-PDFF) for steatosis quantification were introduced with excellent results, but magnetic resonance imaging remains too expensive and time-consuming for routine clinical use [13,18].

In the last two decades, ultrasound (US)-based liver elastography methods have been widely adopted as non-invasive liver fibrosis evaluation tools. Transient elastography (TE) is the most validated US elastography technique and it was the first to be developed for fibrosis evaluation [19]. Subsequently, point shear wave elastography (p-SWE) and two-dimensional shear wave elastography (2D-SWE) have been developed and are now widely available on several commercial US devices. In both cases, liver stiffness (LS) is estimated by shear wave (SW) velocity with the advantage of B-mode imaging [20].

The study of the SW propagation velocity is not the only factor associated with the viscoelastic properties of the liver. Shear wave dispersion (SWD) is a new imaging technique based on 2D-SWE that analyses the frequency-dependent variation in SW velocity, which is strictly related to liver viscosity [21].

In this review, following a brief discussion of the physical principles of SWD, we summarized the pre-clinical and clinical evidence regarding the role of SWD in CLD assessment.

## 2. Physical Principles of SWD

SWs are laterally propagating waves generated by an acoustic radiation force impulse (ARFI) [22]. The characteristics of SWs (such as speed, attenuation, and dispersion) are strictly correlated to the properties of the propagation medium. Dispersion, which is usually less known than SW speed or US attenuation to clinicians and researchers, is defined as the dependence of speed variations on vibration frequency [23]. Viscosity and elasticity are the two parameters that most influence SWs’ properties [24]. Thus, in a nutshell, by analysing the characteristics of SWs we can estimate the viscosity and elasticity of a propagation medium (i.e., liver tissue). 

The liver parenchyma can be considered a viscoelastic system. The majority of US-based ARFI methods, however, ignore dispersion and only calculate effective elasticity. Consequently, information regarding viscosity is lost, and the resulting effective elasticity may be partial [25].

Different viscoelastic models were investigated to describe SWs’ propagation into the liver. The so-called Zener model consists of a spring and dashpot in parallel with a spring, whereas the widely used Voigt model consists of a spring and dashpot in parallel [26]. However, it is unclear which model matches hepatic viscoelasticity measurements best [27,28]. 

According to the Voigt model, the relation between the frequency-dependence of SW speed and medium properties is expressed as

$$C_{s}\left(\omega \right)=\sqrt{\frac{2\left(\mu^{2}+\omega^{2}\eta^{2}\right)}{\rho \left(\mu +\sqrt{\mu^{2}+\omega^{2}\eta^{2}}\right)}}$$

where $C_{s}\left(\omega \right)$ is the SW speed at *ω* frequency of vibration, *ρ* is the density of the medium. *μ* and *η* are the shear elasticity and shear viscosity of the medium, respectively [23]. 

**Figure 1:**
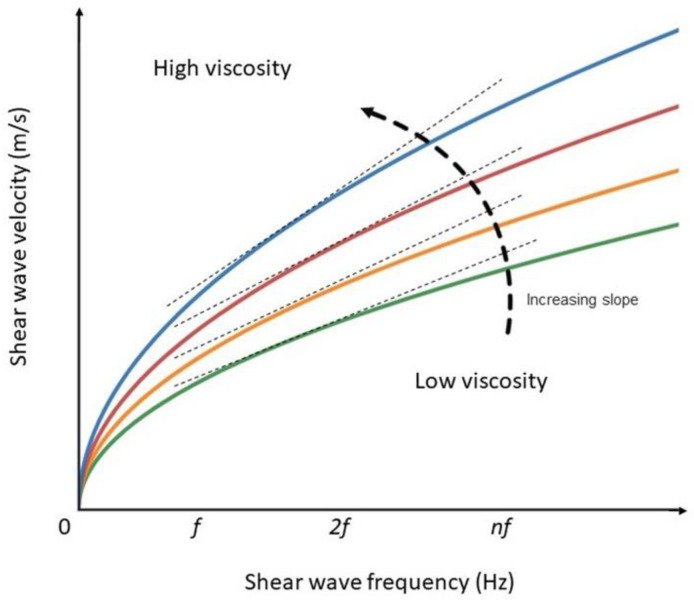
Variability of shear wave viscosity, frequency, and dispersion slope in viscoelastic medium.

When a tissue is dispersive, i.e., has a high viscosity, the speed of SWs increases with frequency, and vice versa; this allows viscosity to be approximated by knowing the speed of SWs at a specific frequency. Considering the relationship between speed and frequency, we can derive the slope of the curve, which is also an indirect measure of viscosity (Figure 1). 

Inflammation, steatosis, and fibrosis, which are dynamic biological phenomena implicated in CLD, change the properties of liver viscoelastic systems and SWD analysis appears as a potential tool for identifying and quantifying these modifications.

## 3. Preclinical Studies

Using rat models with varying degrees of liver damage induced by different amounts of carbon tetrachloride, Sugimoto et al. demonstrated that SW speed and shear wave dispersion slope (SWDS) were significantly higher in rats with higher histological grades of fibrosis and necro-inflammation, respectively. Confirming this, SWDS was independently related to necrosis, in contrast to SW speed which was related to fibrosis [29]. 

In another similar in vivo experiment, Furuichi et al. confirmed that SWDS was higher in rat models with predominant necro-inflammatory liver damage than in models with prevalent fibrotic liver damage with only slight necro-inflammation. Interestingly, splenic SWDS and splenic histologic features were also assessed. Splenic necro-inflammation was absent and splenic SWDS values did not differ significantly between groups. SW speed increased only when splenic fibrosis was documented [30]. Therefore, fibrosis, which modifies viscoelastic properties, appears to affect SW speed, whereas necro-inflammation, which modifies only the viscous component, appears to affect SWDS.

Other biological factors can influence viscosity and, consequently, SWDS. In an ex vivo experiment conducted by Barry et al., SWDS increased in groups with higher triglyceride levels in the liver, indicating that steatosis may also contribute to a viscous component that correlates with dispersion [31].

## 4. Clinical Studies 

SWDS and viscosity are two parameters that can both be referred to as SWD. Currently, commercially available equipment with the SWD estimation function provides the result as SWDS, which is measured in (m/s)/kHz (i.e., by Aplio, Canon, Japan), or as a viscosity index, which is measured in Pa·s (i.e., by the Supersonic Shear Imagine (SSI, France) device using the Vi.Plus software). 

**Figure 2:**
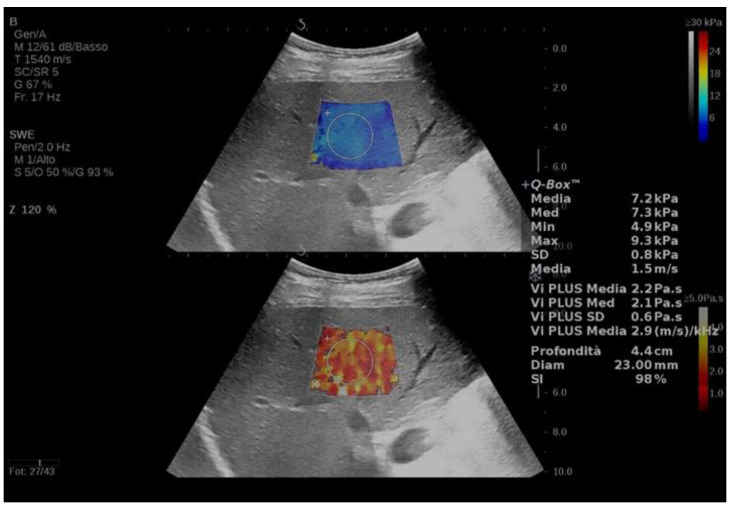
An example of 2D-SWE (upper part of the image) and viscosity (lower part of the image) in a patient with clinically significant liver fibrosis (F2) and mild inflammation (A1) measured with SSI using the Vi.Plus software.

Clinical studies which explore SWD in CLD are summarized in Table 1 (mixed aetiologies) and Table 2 (NALFD aetiology), while an example of viscosity imaging is depicted in Figure 2.

### 4.1. SWD in Healthy Subjects

In 128 healthy children, the mean SWDS (Aplio i800, Canon, Japan) was 11.43 ± 1.75 (m/s)/kHz, while in 32 healthy adults the mean SWDS was 10.24 ± 1.65 (m/s)/kHz [50]. Similarly, in another paediatric healthy population, Cetinic et al. showed a median SWDS (Aplio i800, Canon, Japan) value of 11.7 (range, 9.4–13.7) (m/s)/kHz [33]. SWDS in paediatric liver appears to be slightly higher than in adult liver, which may be related to age-dependent viscoelasticity. Indeed, LS also seems to increase with age as the proportion of collagen increases [51]. In support of this, the SW velocity, which is correlated with collagen content, also increases with height [50]. Therefore, it may be necessary to stratify by age to adequately interpret SWDS values.

In another study utilizing Vi.Plus by SSI, the mean viscosity of 131 healthy adults was 1.59 Pa·s. After multivariate analysis, age and body mass index (BMI) were significantly associated with Vi.Plus [52]. In contrast, Trout et al. found no significant correlation between SWDS and BMI, but a significant positive correlation between SWDS and abdominal wall thickness and median measurement depth [50]. Although the two adult populations were comparable, the differences that arose may not only be attributed to the use of different equipment, but also to differences in exam preparation protocols, as even the intake of water can modify the viscoelastic properties of the liver [53].

### 4.2. SWD in Populations with Different CLD Aetiologies

Populations with CLD of various aetiologies are analysed together in the studies presented in this paragraph. In most of the studies, the reference method was a liver biopsy.

To verify if the use of a viscoelastic model could improve the accuracy of elasticity in fibrosis staging, Chen et al. enrolled 35 patients who underwent biopsy and fibrosis histological assessment. Using a customized device (iU22, Philips, The Netherlands) implemented with SWD US-vibrometery technology, they measured three parameters: Voight elasticity, Voight viscosity, and effective elasticity. The results showed a positive correlation between Voigt elasticity and Voigt viscosity. Voight elasticity, Voight viscosity, and effective elasticity increased with increasing liver fibrosis stage. In the multivariate analysis, Voight viscosity was not significantly associated with fibrosis stage. As Voight elasticity and effective elasticity showed to be more accurate to distinguish F0–F1 from F2–F4 compared to Voight viscosity (area under the curve (AUC) 0.98, 0.95, and 0.86, respectively), the authors concluded that the viscoelastic model does not improve the diagnostic accuracy of the elastic model to classify fibrosis. Interestingly, three patients presented with low elasticity and high viscosity: one of these had acute hepatitis, one had iron overload in hereditary hemochromatosis, and one is not known. Unfortunately, in this study the relationship between inflammatory activity and elasticity parameters was not investigated [54].

In agreement with this study, Wang et al. found a good correlation between SWDS (Aplio i900, Canon, Japan), LS values evaluated with 2D-SWE, and the histological grading of fibrosis in 210 patients undergoing hepatectomy due to hepatocellular carcinoma. Particularly, 2D-SWE showed significantly better accuracy in diagnosing F ≥ 3 and F = 4 compared to SWDS (AUC 0.88 and 0.85 vs. 0.82 and 0.79, respectively), while no significant differences were reported in predicting mild (F ≥ 1) and moderate fibrosis (F ≥ 2). In addition, the necro-inflammatory activity was evaluated and only a weak correlation was found with SWDS. Nonetheless, in multivariate analysis, alanine aminotransferase (ALT), gamma-glutamyl-transferase (γ-GT), total bilirubin, and fibrosis stage emerged as independent factors for SWDS, in contrast to the histological inflammatory activity stage [37]. Recently, the same authors demonstrated a moderate correlation between SWE and SWDS and inflammatory activity in a similar study comprising benign and malignant liver tumours (hepatocellular carcinoma, intrahepatic cholangiocarcinoma, mixed hepatocellular carcinoma-intrahepatic cholangiocarcinoma, and liver metastases), but multivariate analysis confirmed the absence of an association. Interestingly, they described a significantly higher LS and SWDS of liver parenchyma in primary liver cancer compared to other liver tumours [32].

Similarly, Ferraioli et al. found a strong and significant positive correlation between LS values measured by 2D-SWE and SWDS (Aplio i800, Canon, Japan). In a prospective study including 367 patients and using TE as reference method for fibrosis classification, they showed a significant difference between F0-1, F2, and F3-4 for SWDS (9.8, 13.6, and 17.5 (m/s)/kHz, respectively). ALT, which is considered a surrogate marker of liver inflammation, was poorly correlated with SWDS, with nearly 80% of the population having normal ALT values [40].

In a prospective study including 120 patients, Deffieux et al. described a higher viscosity (measured with SSI device) (AUC of 0.72) for elevated inflammatory activity (A ≥ 2). Despite the fact that no statistically significant correlation was found between viscosity and inflammation or steatosis, viscosity values only increased according to the severity of inflammation (2.2, 3, and 4.1 Pa·s for A1, A2, and A3, respectively) [43]. Accordingly, Zhang et al. showed that SWDS (Aplio i900, Canon, Japan) was positively correlated with necro-inflammation and was significantly different among necro-inflammatory categories (*p* = 0.02). The AUC of SWDS was inferior to the AUC of LS or LS plus SWDS in detecting A ≥ 2 (0.64 vs. 0.75 and 0.75, respectively). In a subgroup analysis, considering patients with clinically significant fibrosis (F2-4), SWDS was significantly higher in A2-3 compared to A0-1 (*p* = 0.04) [38].

Another potential application using liver viscosity was evaluated in a prospective study involving 104 liver transplant recipients who underwent liver biopsies for allograft evaluation. The authors (Lee et al.) demonstrated a clear correlation between inflammatory activity and viscosity in this clinical setting. The allograft was considered damaged if acute cellular rejection, cholangitis, the reactivation of viral hepatitis, NASH, or alcohol hepatitis were detected. SWDS (Aplio i900, Canon, Japan) was significantly higher in allograft damage compared to no allograft damage (14.4 (m/s)/kHz vs. 10.4 (m/s)/kHz, *p* ≤ 0.01). The AUC for allograft damage was higher for SWDS than LS (0.86 vs. 0.75, respectively). In a multivariate analysis, only fibrosis was a determinant for allograft LS, while fibrosis and necroinflammatory activity were determinants for SWDS. SWDS provided a better diagnostic performance for allograft damage as compared to the LS value alone, likely because the involvement of inflammation is also reflected [42].

To summarize, SWD and SWE are frequently correlated with each other and with the degree of fibrosis, indicating that both the elastic and viscoelastic models are valid in the assessment of fibrosis. Contrarily, this correlation was not always observed between SWD and necro-inflammatory activity, as expected. The reasons could be different. First, the characteristics of studied populations need to be taken into account. Indeed, we have described a significant relationship between SWD and inflammation in patients with allograft damage [42], while only a weak correlation exist in populations with normal ALT levels [40] or in populations excluding patients with higher levels of inflammatory activity [32,37], suggesting that a correlation between inflammation and SWD probably exists. Secondly, the histologic classification systems for fibrosis, inflammation, and steatosis varied between studies, and the chosen methods may not be suitable for a generalized description of populations with various aetiologies. Thirdly, variations in study protocols regarding the acquisition of elasticity/viscosity measurements and the employed US equipment might also lead to differences in SWD values.

Only one study investigated the relationship between SWD and histopathologic features in a paediatric population. In this study, 32 children with known or suspected liver disease underwent liver biopsy and were classified according to fibrosis, inflammation, and steatosis degree. SWDS (Aplio i800, Canon, Japan) showed a weak positive correlation with the grade of fibrosis (r = 0.4; *p* = 0.02), while it was significantly different between different grades of inflammation (*p* < 0.008) and significantly lower in A0-1 compared to A2-3 (*p* = 0.0028). Furthermore, SWDS was lower in the control group compared to all A > 0 and compared to A0 (*p* < 0.005 and *p* = 0.0008, respectively). Interestingly, children in the A0 group had higher SWDS values than those in the control and A1 groups. This supports the idea that inflammation is not the only factor that influences viscosity [33,55,56].

### 4.3. SWD and NAFLD/NASH

The ideal goal of the non-invasive evaluation of NAFLD would be to identify patients with NASH, which is currently only achieved by evaluating histologic characteristics [57]. So far, some studies have tried to investigate the relationship between SWDS and histologic features in NAFLD patients.

In a prospective study involving 111 patients who underwent liver biopsies for suspected NAFLD, Sugimoto et al. investigated the role of SWDS (Aplio i800, Canon, Japan) in the diagnosis of NASH. The results showed that SWDS significantly increased with the degree of inflammation. Using a cut-off value of 8.5 (m/s)/kHz, SWDS showed a very high accuracy in identifying the presence of lobular inflammation ≥1 with an AUC of 0.95 and a good ability to distinguish inflammation grade ≥ 2 and = 3 with AUCs of 0.81 and 0.85, respectively. Interestingly, when combining SW speed (which represents LS), the attenuation coefficient, and SWDS in a regression model, the total AUC to diagnose NASH was higher than the AUC of each single parameter (0.81 vs. 0.76, 0.71 and 0.70 for SWDS, attenuation coefficient, and SW speed, respectively). Furthermore, SWDS and the attenuation coefficient were significantly influenced by lobular inflammation and steatosis grade, while fibrosis was significantly influenced by SW speed [49]. These results confirmed their previous findings that SWDS was significantly higher in high-grade lobular inflammation [29]. In another study by the same authors, a novel score based on multiparametric US features (LS, attenuation coefficient, and SWDS) was developed in order to identify high-risk NASH patients defined as subjects with NAFLD activity score (NAS) ≥ 4 and fibrosis stage ≥ 2. This newly developed “LAD-NASH score” showed a good diagnostic performance to correctly identify high-risk NASH with an AUC value of 0.86 in the derivation cohort (111 patients enrolled in Japan) and of 0.88 in a separate validation cohort (102 patients from Korea) [48]. By using such a non-invasive score, it may be possible to correctly identify patients requiring treatment for NASH without the need for liver biopsy, similarly to the Fibroscan-AST (FAST) score that is based on the combination of LS measurement by TE (which is related to liver fibrosis) and the controlled attenuation parameter (CAP, which is related to liver steatosis) with factors linked to inflammation (AST) [58].

Recently, Jang et al. conduced a multicentric prospective study in 132 patients undergoing liver biopsy for suspected NASH. The AUCs of SWDS (Aplio i800, Canon, Japan) were 0.86, 0.86, and 0.79 for detection inflammation grades ≥1, ≥2, and =3, respectively. Interestingly, they created another risk score for NASH detection considering attenuation coefficient, SWDS, and LS, which showed a very high performance with an AUC of 0.94 and a sensitivity, specificity, positive predictive value, and negative predictive value of 81%, 96%, 93%, and 88%, respectively. In multivariate analysis, lobular inflammation was the only significant determinant factor for SWDS, steatosis was the only significant factor for the attenuation coefficient, and lobular inflammation and fibrosis stage were the only significant factors for LS [46]. This confirms that SWE, SWDS, and the attenuation coefficient correctly identify distinct components of liver disease severity and support the role of SWDS as reliable tool to identify and graduate inflammation in NAFLD patients.

The triglyceride accumulation In ”epat’cytes alters the viscoelastic properties of the liver parenchyma [21,59]. Can SWDS detect steatosis even if the attenuation coefficient is normal, i.e., in the early stages of hepatic steatosis? In the studies included in this review that used liver biopsy as referenced method, it is not analysed if and how SWDS may change in different histologic steatosis grades. Considering MRI-PDFF as reference method, SWDS showed a higher performance in diagnosing steatosis compared to the attenuation coefficient, although the attenuation coefficient showed the highest correlation with MRI-PDFF [44]. In another study, Vi.Plus results were independently associated with BMI but not ALT [47]. These findings suggest that SWDS may vary in the early stages of NAFLD due to viscoelastic alterations, independently of inflammation, and the accumulation of liver lipids being insufficient to result in US attenuation.

### 4.4. SWD and Viral Hepatitis

To the best of our knowledge, no research has investigated the relationship between SWDS and histologic characteristics in a cohort of patients with only chronic viral hepatitis.

In the previously mentioned study, Deffieux et al. conducted a subgroup analysis of patients with chronic viral hepatitis showing that the AUCs of viscosity were 0.6, 0.83, and 0.65 for inflammation grades A ≥ 1, A ≥ 2, and A = 3, respectively, while SWDS showed lower values of AUC in all cases [43].

In another study, Su et al. evaluated the variation in SWDS (Aplio i800, Canon, Japan) before and after treatment with directly acting antivirals in 122 patients with chronic C hepatitis. SWDS showed a reducing trend after 12 weeks after the end of therapy (11.6 vs. 11.2 (m/s)/kHz), but with no statistically significant relevance [41]. It is noteworthy that the mean SDWS values prior to therapy were higher than the mean values in healthy subjects [52], but lower than the mean values of inflammatory grade 0 in the study by Zhang et al., in which 50% of the population had chronic viral hepatitis [38]. In these three studies, the SWDS was evaluated using the same equipment. This may indicate that variation in SWDS was not detected due to a lower initial level of inflammation.

### 4.5. SWD and Other Specific Aetiologies

In 29 patients with alpha-1 antitrypsin deficiency, SWDS (Aplio i800, Canon, Japan) was higher than the normal value described (14.2 (m/s)/Hz vs. (range 10.3–18.5 (m/s)/Hz vs.) but did not show a correlation with ALT as a necro-inflammation marker [39]. Similar results were shown in 22 patients with primary biliary cholangitis in which the mean SWDS (Aplio i800, Canon, Japan) value was 13.9 (m/s)/kHz (range, 11.6–21 (m/s)/kHz) and was significantly correlated with ALP and γ-GT [35]. Further investigations are needed to correlate SWD to histologic features in these specific aetiologies of CLD.

Another potentially useful application of liver SWD might be found in patients with heart failure and haemodynamic alterations, given that liver viscosity could be influenced by sinusoidal congestion.

SDWS (Aplio i900, Canon, Japan) showed a gradual increase from an early stage to advanced stage of chronic heart failure, while LS was demonstrated to be remarkably elevated only in advanced stages with liver function test abnormalities [60]. In another study, the dichotomic classification of patients according to a SWDS cut-off ≥10 (m/s)/kHz was associated with high cardiac event rates (hazard ratio of 2.84) [61]. Consequently, SWDS could be a potential tool to early detect liver injury in chronic heart failure and to provide prognostic information. Finally, in Fontan-associated liver disease, long-term passive sinusoidal congestion causes liver damage with time-dependent progression from hepatic congestion to fibrosis without portal hypertension to cirrhosis after the Fontan procedure [62]. In 30 adult patients who had undergone the Fontan procedure in their medical history, central vein pressure was significantly correlated with SWDS (Aplio i800, Canon, Japan) while it was not correlated with LS. Furthermore, SWDS tended to increase with time after surgery, suggesting that SWDS might be a helpful indicator to monitor these patients [34].

### 4.6. SWD in Advanced CLD

Given the fact that inflammation increases intrahepatic vascular resistance and enhances portal hypertension, SWD may play a role in determining the risk of portal-hypertension-related complications in advanced CLD; however, portal-hypertension-related complication such as ascites may impact the reliability of SWD measurements [63].

Sun et al. investigated the association between SWDS (Aplio i900, Canon, Japan) and variceal haemorrhage (VH) in 65 cirrhotic patients. Patients without any clinical symptoms of gastrointestinal (GI) bleeding were classified as non-VH, whereas patients with suspected GI bleeding underwent upper-GI endoscopy and were classified as VH if signs of bleeding were present. SWDS was significantly higher in the VH group compared to the non-VH group (17.04 vs. 15.17 (m/s)/kHz, *p* < 0.001). Multivariate analysis revealed that SWDS, splenic diameter, and ascites were independently associated with VH, and a model based on these three factors distinguished VH from non-VH with excellent performance and maximal negative predictive value. Interestingly, LS was not different between the two groups, suggesting that inflammation plays an important role in the progression of portal hypertension independently of fibrosis [36].

Recently, Hirooka et al. demonstrated a correlation between splenic and hepatic SWDS (Aplio i900 and i800, Canon, Japan) and hepatic venous pressure gradient (HVPG). The AUC of splenic SWDS for HVPG >12 mmHg and high-risk esophagogastric varices were greater than those of hepatic SWDS (0.79 and 0.83 vs. 0.65 and 0.61, respectively), although there was a better performance of splenic SW speed compared to SWD in both cases (AUC 0.91 and 0.89, respectively) [64].

## 5. Conclusions

In recent years, non-invasive techniques, such as TE, MRE, and MRI-PDFF, have been developed and validated for the evaluation of fibrosis and steatosis. In contrast, liver biopsy remains the only method for evaluating inflammation. The assessment of SWD based on the viscoelastic properties of the liver parenchyma could be a potential non-invasive method for assessing inflammation. Prospectively, multiparametric US seems to be the most promising tool for the non-invasive evaluation of all pathogenetic components of CLDs. Nonetheless, further investigations on larger scales that focus on specific aetiology groups, using liver biopsy as the reference method with uniform and appropriate histological scores for the assessment of inflammation, are warranted.

## Figures and Tables

**Table 1:** Clinical studies utilising shear wave dispersion (SWD) for CLD with mixed aetiologies.

Author (Year)	Study Design (N)	Aetiology of CLD	Reference Method	Population	US Device	Parameter (Unit)	Mean SWD Values (SD) or Median [IQR]	Main Results
Wang et al. (2023) [32]	Prospective (174)	Mixed aetiologies	Histology	Patients undergoing hepatectomy for liver tumours	Canon, Aplio i900	SWDS (m/s)/kHz	F0: 10.2 F1: 11.7 F2: 13.3 F3: 14.1 F4: 18.6	SWDS was significantly higher in patients with primary liver cancer and showed good correlation with fibrosis stage (r = 0.87); moderate correlation with necro-inflammatory activity (r = 0.55); no correlation with steatosis (r = 0.13).
Cetinic et al. (2022) [33]	Prospective (32)	Mixed aetiologies	Histology	Children with known or suspected liver disease undergoing liver biopsy	Canon, Aplio i800	SWDS (m/s)/kHz	A0: 13.1 [8.4–17.2] A1: 13.6 [11.3–17.2] A2:16.1 [10.7–24.2] A3: 15.8 [15.3–16.3]	SWDS was significantly different between grades of inflammation.
Nagasawa et al. (2022) [34]	Cross-sectional (30)	Fontan-associated liver disease	CT	Fontan-associated liver disease undergoing liver US and CT	Canon, Aplio i800	SWDS (m/s)/kHz	Controls: 9.3 F01: 12.5 [10.3–14.9] F ≥ 2: 17.6 [15.5–20.7]	SWDS was significantly different between the three groups. SWDS significantly correlated with central venous pressure (r = 0.53).
Schulz et al. (2022) [35]	Cross-sectional (22)	Primary biliary cholangitis	-	Patients undergoing liver US	Canon, Aplio i800	SWDS (m/s)/kHz	All patients: 13.9 [11.6–21]	SWDS was significantly correlated with ALP (r = 0.54) and γ-GT (r = 0.49).
Sun et al. (2022) [36]	Prospective (65)	Mixed aetiologies	-	Cirrhotic patients undergoing upper gastrointestinal endoscopy	Canon, Aplio i900	SWDS (m/s)/kHz	Variceal haemorrhage group: 17.0 (1.45) Non-variceal haemorrhage group: 15.2 (1.88)	SWDS was an independent risk factor for variceal haemorrhage and was significantly higher in variceal haemorrhage group.
Wang et al. (2022) [37]	Prospective (210)	Mixed aetiologies	Histology	Patients undergoing hepatectomy for hepatocellular carcinoma	Canon, Aplio i900	SWDS (m/s)/kHz	F0: 11.8 (0.39) F1: 12.3 (0.91) F2: 13.6 (0.32) F3: 15.2 (0.44) F4: 17.4 (0.34)	SWDS showed a good correlation with fibrosis stage (r = 0.58); weak correlation with necro-inflammatory activity (r = 0.28); no correlation with steatosis (r = 0.17).
Zhang et al. (2022) [38]	Prospective (159)	Mixed aetiologies	Histology	Patients undergoing liver biopsy for CLD evaluation	Canon, Aplio i900	SWDS (m/s)/kHz	F0: 12.3 [11.3–13.9] F1: 13.1 [12–14.7] F2: 14.2 [12.4–16.6] F3: 15.7 [14.2–18.7] F4: 16.7 [13.6–18.4] A0: 12.3 [10.6–14.5] A1: 13.1 [12–15.4] A2: 14.1 [12.7–17] A3: 16.6 [13.4–17.5] S0: 13.9 [12.2–16] S1: 12.6 [11–15.2] S2: 12.6 [10.4–13.5]	SDWS was significantly different among necro-inflammatory stages and fibrosis stages and significantly higher in A2-3 vs. A0-1.
Schulz et al. (2021) [39]	Cross sectional (29)	Alpha1-antitrypsin deficiency	-	Patients undergoing liver US	Canon, Aplio i800	SWDS (m/s)/kHz	All patients: 14.2 [10.3–18.5]	SWDS was significantly correlated with spleen diameter and platelet count, while no correlation was found with ALT and AST.
Ferraioli et al. (2021) [40]	Cross-sectional (367)	Mixed aetiologies	Transient elastography	Patients underwent liver stiffness measurement	Canon, Aplio i800	SWDS (m/s)/kHz	F0-1: 9.8 [8.8–10.8] F2: 13.6 [12–14.8] F3-4: 17.5 [13.7–20.5]	SWDS was significantly different between F0-1 vs. F2 and F0-1 vs. F3-4. SWDS showed poor correlation with ALT (r = 0.18).
Su et al. (2020) [41]	Retrospective (122)	Viral hepatitis	-	Patients with chronic C hepatitis treated with direct antiviral agents	Canon Aplio i800	SWDS (m/s)/kHz	Baseline: 11.6 [9.3–13.9] 12 weeks after end of therapy: 11.2 [9.2–13.3]	SWDS showed no significant modification after antiviral therapy.
Lee et al. (2019) [42]	Prospective (104)	Mixed aetiologies	Histology	Liver transplanted patients undergoing biopsy for allograft evaluation	Canon, Aplio i900	SWDS (m/s)/kHz	Allograft damaged: 14.4 [12.3–16.5] Allograft without damage: 10.4 [8.9–13]	SWDS was significantly higher in patients with allograft damage. Fibrosis and inflammatory activity were independently related to SWDS.
Deffieux et al. (2015) [43]	Prospective (120)	Mixed aetiologies and subgroup analysis in viral hepatitis	Histology	Patients with CLD undergoing liver biopsy	Supersonic Imagine, Aixplorer	Viscosity (Pa·s)	F0: 2 [0.8] F1: 2.3 [0.7] F2: 2.6 [0.5] F3: 2.7 [1.9] F4: 3.7 [2.5] A0: 2.2 [0.9] A1: 2.2 [0.8] A2: 3 [1.3] A3: 4.1 [2.4] S0: 2.2 [0.9] S1: 2.7 [1.3] S2: 2.7 [1.5] S3: 1.9 [0.3]	Viscosity showedfor F4 an AUC of 0.81 in all patients and 0.87 in viral hepatitis; for A ≥ 2 an AUC of 0.72 in all patients and 0.83 in viral hepatitis; for S ≥ 1 an AUC of 0.63 in all patients and 0.64 in viral hepatitis.

**Abbreviations**: N, number of patients; SWD, shear wave dispersion; SD, standard deviation; IQR, interquartile range; A, inflammatory activity stage; ALT, alanine aminotransferase; ALP, alkaline phosphatase; AST, aspartate aminotransferase; AUC, area under the curve; CLD, chronic liver disease; CT, computed tomography; F, fibrosis stage; γ-GT, Gamma-Glutamyl-Transferase; S, steatosis stage; SWDS, shear wave dispersion slope; US, ultrasound.

**Table 2:** Clinical studies utilising shear wave dispersion (SWD) for non-alcoholic fatty liver disease (NAFLD).

Author (Year)	Study Design (N)	Reference Method	Population	US Device	Parameter (Unit)	Mean SWD Values (SD) or Median [IQR]	Main Results
Platz Batista da Silva et al. (2023) [44]	Retrospective (15)	MRI-PDFF	Patients undergoing US and MRI-PDFF for hepatic steatosis	Canon, Aplio i800	SWDS (m/s)/kHz	All patients: 16.5 (4.58)	SWDS showed moderate correlation with MRI-PDFF steatosis (r = 0.55) and good accuracy to diagnose steatosis (AUC 0.73, cut-off 18.5).
Gao et al. (2022) [45]	Prospective (21)	MRI-PDFF	Patients undergoing US and MRI-PDFF for hepatic steatosis	Canon, Aplio i800	SWDS (m/s)/kHz	Steatosis group: 12.33 (0.87) No steatosis group: 10 (1.53)	SWDS was significantly higher in patients with liver steatosis compared to patients with normal liver.
Jang et al. (2022) [46]	Prospective (132)	Histology	Patients undergoing biopsy for suspected NASH	Canon, Aplio i800	SWDS (m/s)/kHz	-	SWDS showed for A ≥ 1 an AUC of 0.86, cut-off 10.8; for A ≥ 2 an AUC of 0.86, cut-off 11.4; for A3 an AUC of 0.79, cut-off 11.6.
Popa et al. (2021) [47]	Cross-sectional (204)	-	Patients undergoing liver US	Supersonic MACH 30 with Vi.Plus	Viscosity (Pa·s)	All patients: 1.8 (0.83)	BMI and liver stiffness measured with 2D shear wave elastography were independently associated with Vi.Plus.
Sugimoto et al. (2021) [48]	Retrospective (111 (derivation cohort) and 102 (validation cohort))	Histology	Patients undergoing biopsy for suspected NAFLD	Canon, Aplio i800	SWDS (m/s)/kHz	Derivation cohort: 11.17 (2.24) Validation cohort: 11.53 (2.62)	A model based on stiffness, dispersion, and attenuation (LAD-NASH score) was able to identify high-risk NASH patients with high accuracy (AUC 0.86).
Sugimoto et al. (2020) [49]	Prospective (111)	Histology	Patients undergoing biopsy for suspected NAFLD	Canon, Aplio i800	SWDS (m/s)/kHz	All patients: 11.17 (2.24)	SWDS showed for A ≥ 1 an AUC of 0.95, cut-off 8.5; for A ≥ 2 an AUC of 0.81, cut-off 9.9; for A3 an AUC of 0.85, cut-off 12.5.

**Abbreviations**: N, number of patients; SWD, shear wave dispersion; SD, standard deviation; IQR, interquartile range; AUC, area under the curve; BMI, body mass index; MRI-PDFF, magnetic resonance imaging–proton density fat fraction; NAFLD, non-alcoholic fatty liver disease; NASH, non-alcoholic steatohepatitis; SWDS, shear wave dispersion slope; US, ultrasound.

## Data Availability

Data sharing not applicable.
